# REM sleep behaviour disorder: the importance of early identification in primary care

**DOI:** 10.3399/bjgp23X731721

**Published:** 2022-12-30

**Authors:** Samantha Bramich, Kumud Verdi, Katharine Salmon, Alastair Noyce, Jane Alty

**Affiliations:** Wicking Dementia Research and Education Centre, University of Tasmania, Hobart, Australia.; Hillfoot Surgery, West Yorkshire, UK.; South Hobart, Tasmania, Australia.; Wolfson Institute of Population Health, Queen Mary University of London, London, UK.; Wicking Dementia Research and Education Centre, University of Tasmania, Hobart, Australia; neurologist, Royal Hobart Hospital, Hobart, Tasmania, Australia; honorary consultant neurologist, Leeds Teaching Hospitals NHS Trust, Leeds, UK.

## REM SLEEP BEHAVIOUR DISORDER AND WHY IT MATTERS?

REM (rapid eye movement) sleep behaviour disorder (RBD) is characterised by vivid dreams and dream enactment behaviour such as kicking, shouting, and punching.^1^ It can result in injuries to the person with RBD and their bed partner, and is associated with reduced quality of life and severely disrupted sleep.

It is important to recognise potential RBD because it is also strongly associated with neurodegenerative disease (NDD): RBD affects approximately 50% of people with Parkinson’s disease (PD), 80% of people with dementia with Lewy bodies (DLB), and almost 100% of people with multiple system atrophy (MSA).^2^^,^^3^ RBD is also an early sign of neurodegenerative disease and can occur in otherwise healthy individuals when it is known as idiopathic or isolated RBD (iRBD). This isolated form is associated with an 80–90% risk of progression to an NDD, such as PD or DLB, within 10 years of first diagnosis.^3^ iRBD commonly presents in males >50 years and is estimated to occur in 2% of the general population.^4^ Thus, identifying iRBD provides a rare opportunity to modify future risk of neurodegeneration and there are anticipated opportunities for patients with iRBD to enrol in neuroprotective drug trials to prevent progression to an overt NDD.

## WHY DOES RBD OCCUR?

When we sleep, we pass through four distinct stages. During the stage of sleep most associated with dreaming, REM sleep, the eyes move around quickly but all other skeletal muscle tone is lost (atonia) and thus limbs become floppy; this effectively causes paralysis of our bodies during dreaming. However, in RBD the brainstem system that produces atonia is faulty, possibly damaged by early pathology, and people can therefore physically act out their dreams.^4^

## WHEN TO SUSPECT RBD?

Most people with RBD are unaware of it, but some present to their GP reporting frequent vivid dreams, unexplained injuries (related to thrashing into a wall or furniture when dreaming), or falling out of bed. More commonly, presentation to a GP is initiated by a bed partner who notices kicking and punching movements during sleep.^1^ Unfortunately, it can be challenging for clinicians to disentangle such symptoms from other sleep disorders that have similar symptoms, and therefore RBD is generally under-recognised (Table 1).^1^

**Table 1.** Sleep disorders that present with symptoms similar to those seen in REM sleep behaviour disorder (RBD)

**Sleep disorder**	**Symptoms similar to RBD**
Obstructive sleep apnoea	Unpleasant dreams Body movements due to apnoeas Frequent nocturnal awakenings
Sleep walking	Complex actions performed while asleep
Night terrors	Talking/yelling during sleep
Periodic limb movement disorder	Vigorous limb movements during sleep

## WHAT SHOULD I DO IF I SUSPECT RBD? THE ROLE OF THE GP

**Figure 1.**
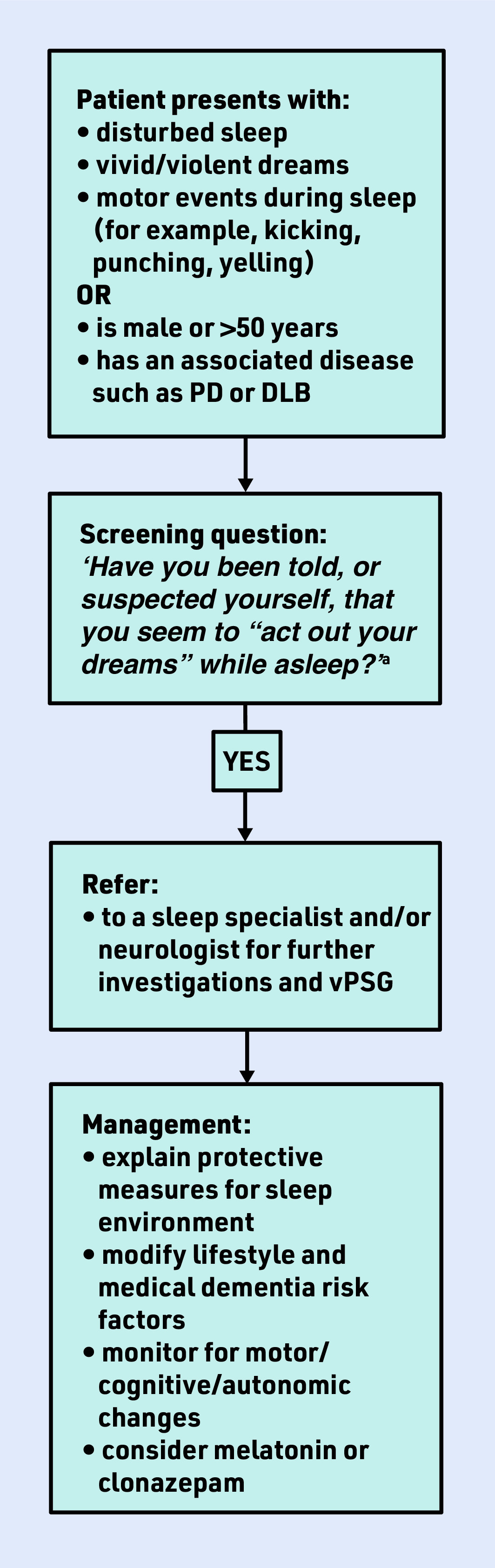
*RBD management flow chart.* *^a^ Caution: if the patient DOES NOT have a bed partner, consider onward referral even if their response is NO to the single screening question but they do have a history suggestive of RBD. DLB = dementia with Lewy bodies. PD = Parkinson’s disease. RBD = REM sleep behaviour disorder. vPSG = video polysomnography.*

GPs have a crucial role in identifying the symptoms of RBD (and other sleep disorders; see National Institute for Health and Care Excellence guidance^5^) and referring patients on to a neurologist or sleep specialist for further assessment. The International RBD Study Group state that an overnight sleep study (video polysomnography [vPSG]) in a sleep laboratory is mandatory for the identification of RBD.^6^ A neurologist may also assess cognitive and motor symptoms to determine any current evidence of a recognisable NDD. In the absence of an overt NDD, diagnosis of iRBD can lead to treatment (associated with improved quality of life), as well as opportunities for risk modification and clinical trial recruitment that hold potential to slow or even stop progression to NDD (Figure 1). GPs also have a key role in addressing medical and lifestyle dementia risk factors, such as hypertension, physical inactivity, and smoking in this group who are at high risk for developing NDDs.

## A SINGLE SCREENING QUESTION

A validated single screening question for ‘probable’ RBD is: *‘Have you been told, or suspected yourself, that you seem to “act out your dreams” while asleep (for example, punching, flailing your arms in the air, making running movements, etc.)?’*^7^ An answer of ‘yes’ should prompt a referral for further investigations. The diagnostic accuracy of this question is 94% sensitivity/87% specificity (sample *n* = 484, ∼70% male, mean age 65 years) when compared with the gold standard of vPSG;^7^ however, this should be used only as an initial screening for onward referral. It is also important to recognise that people without bed partners are far more likely to respond ‘no’ to this question, and a history suggestive of RBD should still prompt referral.

## ARE THERE ANY RISK FACTORS?

Keeping in mind that some people with RBD will already have an NDD, it is important to consider general safety, especially around driving. Visual hallucinations should be directly asked about (as they are common in DLB, for example) and, if present, the patient should be told to stop driving and inform their relevant driving authority. Prolonged antidepressant use may occasionally trigger iRBD, particularly in those taking selective serotonin reuptake inhibitors, selective norepinephrine reuptake inhibitors, and tricyclic antidepressants.^8^ A dose reduction or drug switch of antidepressant drug class should be considered when investigating symptoms of iRBD.

## HOW TO MANAGE SYMPTOMS OF RBD

Two recommended medications to be given at night are clonazepam and melatonin. A recent review found that clonazepam (given at doses ranging from 0.125 to 3 mg) improved symptoms in about two-thirds of patients, but should be used cautiously in older adults, as it can cause morning drowsiness, confusion, and falls. Melatonin is considered a safer option with fewer side effects and, when given at doses ranging from 2 to 6 mg, has been found to improve symptoms in a third of RBD cases.^8^ Sleeping environment modifications are also helpful to minimise risk to people with RBD and their bed partners, including sleeping in a separate room, using pillow barricades, removing potentially dangerous items from the room, or sleeping on a mattress on the floor.^1^

## CAN IRBD BE STOPPED FROM PROGRESSING TO A NEURODEGENERATIVE DISORDER?

This is the central question of numerous research studies, including clinical drug trials, as there is a rare opportunity for intervention up to 10 years *before* degeneration of the brain. In 2020, the *Lancet* Commission provided strong evidence that 40% of all dementia cases are attributable to 12 modifiable risk factors, such as excessive alcohol consumption, hypertension, and smoking,^9^ but currently it is unclear whether the trajectory of iRBD can be changed.

While there remains uncertainty around iRBD and its association with different types of NDD, it is clear that iRBD is a prodrome of neurodegeneration. This means there is an opportunity for dementia risk reduction through managing potentially modifiable dementia risk factors. GPs therefore play a vital role in recognising potential iRBD and applying preventive medicine approaches with the aim of trying to alter iRBD trajectories. Furthermore, GPs will play a similarly vital role in timely referral to specialist settings when cognitive impairment and/or parkinsonism emerge.
